# Coherent solvus of disordered alkali feldspar: experiment, atom probe tomography and thermodynamic model

**DOI:** 10.1007/s00410-024-02150-z

**Published:** 2024-06-06

**Authors:** D. Heuser, R. Dubosq, E. Petrishcheva, G. Bian, C. Rentenberger, C. L. Lengauer, B. Gault, G. Habler, R. Abart

**Affiliations:** 1 Department of Lithospheric Research, University of Vienna, Vienna, Austria; 2 Department Microstructure Physics and Alloy Design, Max Plank Institut für Eisenforschung GmbH, Düsseldorf, Germany; 3 Department of Earth Science, Utrecht Univeristy, Utrecht, The Netherlands; 4 Physics of Nanostructured Materials, University of Vienna, Vienna, Austria; 5 Department of Mineralogy and Crystallography, University of Vienna, Vienna, Austria

**Keywords:** Alkali feldspar, Exsolution experiments, Coherent solvus, Alkali feldspar solid solution, Atom probe tomography

## Abstract

At temperatures above about 600 °C, alkali feldspar forms a continuous solid solution between the Na and K end members. Towards lower temperatures a miscibility gap opens, and alkali feldspar of intermediate composition exsolves, forming an intergrowth of relatively more Na-rich and K-rich lamellae. During exsolution, the crystal structure usually remains coherent across the lamellar interfaces, a feature that may be preserved over geological times. Due to the compositional dependence of the lattice parameters, coherent intergrowth requires that the lamellae are elastically strained. The associated elastic strain energy counteracts exsolution, and the solvus delimiting the misciblity gap for coherent intergrowth lies below the solvus for strain free phase equilibria. To determine the coherent solvus, homogeneous gem quality alkali feldspar of intermediate composition was annealed at conditions falling into the two-phase region of the phase diagram. Thereby a coherent intergrowth of approximately 10–20 nanometers wide lamellae was produced. Lamellar compositions were determined with atom probe tomography defining points on the coherent solvus. In parallel, the coherent solvus was calculated using a thermodynamic mixing model calibrated on the same alkali feldspar as used for the exsolution experiments and accounting for the elastic strain energy associated with coherent lamellar intergrwoth. The experimentally determined and the calculated coherent solvus are in excellent agreement indicating that phase equilibria in coherent lamellar intergrowth of alkali feldspar are adequately described, providing a sound basis for the interpretation of phase relations in coherently exsolved alkali feldspar.

**Supplementary Information:**

The online version contains supplementary material available at 10.1007/s00410-024-02150-z.

## Introduction

Feldspar is the most abundant mineral in the Earth’s crust comprising about 50% of its volume. In terms of major element compositions, feldspar may be described as a solid solution of the $\hbox {CaAl}_2\hbox {Si}_2\hbox {O}_8$ (anorthite), $\hbox {NaAlSi}_3\hbox {O}_8$ (albite) and $\hbox {KAlSi}_3\hbox {O}_8$ (K-feldspar) end members. The Ca and Na end members, and the Na and K end members show good miscibility forming the plagioclase and the alkali feldspar solid solutions, respectively. In contrast, the Ca and K end members show rather limited mutual solubility giving rise to a pronounced miscibility gap along the anorthite - K-feldspar join.

In this study, we address the alkali feldspar solid solution, which shows continuous miscibility between the Na and K end members above about 600 °C, depending on pressure. Towards lower temperatures, a miscibility gap opens giving rise to a two-phase region in the phase diagram that widens with decreasing temperature. The two-phase region is delimited by the so-called *solvus* curve. When cooling from the high temperatures of magmatic or metamorphic crystallisation, alkali feldspar of intermediate composition tends to exsolve, typically forming an intergrowth of relatively more Na-rich and K-rich lamellae. Exsolution occurs by the segregation of $\hbox {Na}^+$ and $\hbox {K}^+$ implying uphill diffusion of these cations on the alkali sites, while the framework of corner sharing $\hbox {AlO}_4$ and $\hbox {SiO}_4$ tetrahedra remains largely unchanged. In particular, the tetrahedral framework remains coherent across lamellar interfaces (Yund and Davidson 1978; Yund 1983; Brown and Parsons 1984; Abart et al. 2009b) and coherency may be preserved over geological times. The lattice parameters of alkali feldspar show considerable compositional dependence (Kroll et al. 1986; Angel et al. 2012). More specifically, the lattice parameters increase with increasing *X*, where $X = n_\text {K}/(n_\text {K} + n_\text {Na})$ is the site fraction of K on the alkali site, and $n_\text {K}$ and $n_\text {Na}$ are atomic concentrations. As a consequence, during chemical segregation the lattice of what becomes a Na-rich lamella shrinks and the lattice of what becomes a K-rich lamella expands. To compensate for the resulting lattice mismatch at the boundaries between Na-rich and K-rich lamellae, both types of lamellae need to be elastically strained so that the two lattices match and the crystal structure remains coherent at the lamellar interfaces. The resulting elastic strain energy constitutes a positive contribution to the Gibbs energy, making the free energy of coherent intergrowth less negative than the free energy of strain free intergrowth with similar phase content and composition. The elastic strain energy associated with coherent lamellar intergrowth thus counteracts exsolution. As a consequence, the two-phase region in the alkali feldspar phase diagram is smaller for coherent than for strain-free intergrowth, and the coherent solvus lies below the strain-free solvus (Robin 1974; Sipling and Yund 1976; Yund 1983; Abart et al. 2009b; Petrishcheva and Abart 2009; Petrishcheva et al. 2023). Coherent lamellar intergrowth has been observed in perthites, in particular, it is typical in crypto- and micro-perthites (Laves 1952; Brown and Parsons 1984; Abart et al. 2009b; Petrishcheva et al. 2020). During recrystallisation or hydrothermal overprint, coherency of the crystal structure across lamellar boundaries may be lost. In this case, the lamellae become strain free and develop compositions given by the strain free solvus (Yund 1983; Abart et al. 2009b).

Exsolution, subsequent lamellar coarsening and the compositional evolution of the exsolution lamellae with decreasing temperature require interdiffusion of Na and K. Diffusion is a thermally activated process, and at some stage during cooling, Na-K interdiffusion becomes so sluggish that the compositions and widths of the lamellae are essentially frozen. The compositions and widths of the lamellae in a perthite are petrographic observables from which the geological cooling history can be inferred (Abart et al. 2009b; Petrishcheva and Abart 2009, 2012). Extracting cooling rates from exsolved alkali feldspar requires that the exact shape and position of the solvus and the underlying Na-K interdiffusion are known. In this study, we focus on the solvus for coherent intergrowth of Na-rich and K-rich lamellae of alkali feldspar, which are typically produced during the cooling induced exsolution of initially homogeneous alkali feldspar of intermediate composition.

In previous studies, the strain free solvus for alkali feldspar was determined experimentally from two-feldspar phase relations for disordered (Orville 1963; Luth and Tuttle 1966; Smith and Parsons 1974; Lagache and Weisbrod 1977) and ordered (Bachinski and Müller 1971) alkali feldspar, and it was calculated from calorimetric data (Haselton et al. 1983; Fuhrman and Lindsley 1988; Hovis et al. 1991; Hovis 2017; Heuser et al. 2024). The coherent solvus was previously determined experimentally (Tullis 1975; Sipling and Yund 1976) and also was calculated from thermodynamic data (e.g. Robin 1974; Tullis and Yund 1979; Petrishcheva et al. 2023). Experimentally produced coherent alkali feldspar exsolution lamellae are usually less than 100 nm thick, which has hitherto impeded in-situ chemical analysis. Lamellar compositions were derived indirectly based on measurement of the lattice parameters by X-ray diffraction (XRD). Noting that along a coherent interface, the crystal structures of both the Na-rich and the K-rich lamellae must be strained to maintain coherency, lamellar compositions were calculated from the measured lattice strain (Robin 1974; Tullis 1975; Tullis and Yund 1979; Yund 1983) relative to the known compositionally dependent lattice parameters of strain-free alkali feldspar (Orville 1967; Kroll et al. 1986; Angel et al. 2012). This calculation hinges on a model for the elastic strain and thus on elastic constants for alkali feldspar. The elastic constants are well constrained for compositions close to the Na and K end members (Waeselmann et al. 2016), but they are poorly known for alkali feldspar of intermediate composition, adding some uncertainty to the indirect determination of lamellar compositions. Moreover, in earlier studies simplifying assumptions were made for calculating the elastic strain associated with coherent lamellar intergrowth (Robin 1974; Williame and Brown 1974), which were relaxed in a recent work by Petrishcheva et al. (2023) so that an adequate model for elastic strain is available. Application of this model to experimentally exsolved alkali feldspar, where lamellar compositions were directly analysed, is, however, pending.

In this study, we used atom probe tomography (APT) for analysing the compositions of exsolution lamellae produced by annealing initially homogeneous gem-quality alkali feldspar at conditions within the two-phase region of the phase diagram. APT provides chemical analyses with nanometre spatial resolution, thus enabling compositional analysis of 10 to 20 nm wide lamellae (Gault et al. 2021). The compositions obtained in this APT study from samples annealed at different temperatures define the first directly measured coherent solvus for alkali feldspar. Coherent intergrowth was verified by powder X-ray diffraction (pXRD) and by selected area electron diffraction (SAED) in transmission electron microscopy (TEM). In parallel, the coherent solvus was calculated based on a thermodynamic mixing model that had been calibrated by Heuser et al. (2024) on the same feldspars as used for the exsolution experiments and using the strain model of Petrishcheva et al. (2023). Since both the calibration of the thermodynamic mixing model and the exsolution experiments were performed with the same feldspars, the results are directly comparable. Excellent agreement between the experimentally determined and the calculated coherent solvus indicates that our model describes phase equilibria in coherent lamellar intergrowth adequately and provides a sound basis for the thermodynamic analysis of phase equilibria in coherent perthitic intergrowth.

## Methods

### Sample material

Two K-rich natural gem-quality alkali feldspars were used as starting materials: sanidine from Itrongay, Madagascar^1^ (M) and from Volkesfeld in the Eifel, Germany (V). The sample material is identical to M and V described in Heuser et al. (2024), and t *X* he reader is referred to this article for the details of the crystallography and chemistry of these feldspars. Both feldspars are homogeneous and devoid of any sign of exsolution in powder X-ray diffraction (pXRD) analyses and in scanning electron microscope (SEM) images (see Supplementary Information, Fig. S3). Both feldspars have space group C2/m. The state of Al-Si order of the starting material as derived from the measured lattice parameters and the equations for monoclinic feldspar given by Kroll and Ribbe (1987) is characterised by $2t_1=0.689$ for M and by $2t_1=0.611$ for V, where $t_1$ represents the proportion of the $\hbox {Al}^{3+}$ on the two $\hbox {T}_1$ sites; $2t_1=0.5$ represents fully disordered and $2t_1=1$ fully ordered Al-Si. Both feldspars are K-rich with compositions of $X = 0.94$ (M) and 0.83 (V), see S1. For minor and trace element compositions please refer to S3 in the Supplementary Information. Centimetre sized specimen were crushed and sieved, and the 63−100 $\mu m$ fraction was used for the exsolution experiments.

### Na-K exchange experiment

Feldspar powder was mixed with NaCl and KCl salt (S2), where the amounts were chosen so that a 40:1 molar ratio between alkali cations in the salt relative to alkali cations in the feldspar was obtained. The feldspar salt mixture was sealed in an evacuated quartz-glass tube, heated in a muffle furnace and kept at $900^\circ \hbox {C}$ at approximately atmospheric pressure for 35 days. At this temperature the salt is molten ensuring perfect contact with the feldspar grains allowing for efficient cation exchange between salt melt and feldspar. The proportions of NaCl and KCl in the salt were chosen so that the feldspar compositions were shifted to $X = 0.44$ during cation exchange (Tab. S2). For this composition, the solvus was expected to reach the highest temperature. After annealing, the samples were quenched in water, and the powder was rinsed several times with purified water to remove the salt. High contrast BSE images produced from polished grain mounts were used to check for compositional homogeneity of the samples (Fig. S3). After cation exchange, electron probe microanalysis (EPMA) yielded $X = 0.44$ for M and $X = 0.46$ for V.

### Exsolution experiments

The powders that had been shifted to intermediate compositions were used for the exsolution experiments. For each experiment, about 15 $mg$ of feldspar powder were sealed in an evacuated quartz-glass tube and annealed at four temperatures ranging from $440^\circ \hbox {C}$ to $560^\circ \hbox {C}$ in muffle furnaces for up to 256 days (see Tab. 1). The annealing temperatures were chosen so that they lie within the two-phase region of the phase diagram and the maximum temperature was chosen close to the expected maximum temperature of the coherent solvus. The temperature was measured with an N-type thermocouple placed 2-4 cm above the samples. After annealing, the samples were quenched in water to room temperature. Each sample powder was mounted in phenolic resin with graphite filler, and the grain mounts were polished for further analyses.

**Table 1:** Overview of exsolution experiments

T [°C]	Days at T					
440		16	32	64	128	
480	4	16	32	64	128	
520	4	16	32	64	128	256
560	4	16	32	64		

### Powder X-ray diffraction

Powder X-ray Diffraction (pXRD) was performed on the untreated feldspars and on the material that was shifted to intermediate compositions before and after the exsolution experiments for all temperatures using the run products from the 64 days annealing experiments. For XRD analysis, the feldspar powders were milled to smaller grain sizes to minimise textural effects. The pXRD measurements were done on a Philips PW3020 diffractometer at the Faculty of Geosciences, Geography and Astronomy (FGGA), University of Vienna (Austria), using CuK$\alpha$ radiation. The TOPAS-6 software was used for Rietveld refinements to extract unit cell parameters.

### Electron probe microanalysis

Electron Probe Microanalysis (EPMA) was applied to determine the major element compositions of the starting materials and of the specimens, which were shifted to intermediate compositions, in order to also check for their chemical homogeneity. The measurements were done with a Cameca SX Five electron probe microanalyser at the FGGA, University of Vienna, Austria. An acceleration voltage of 15kV and a beam current of 20nA were applied. The beam was defocused to a diameter of 6$\mu m$ to minimise loss of Na by evaporation. Natural and synthetic silicate and oxide standards were used for calibration.

### Electron backscatter diffraction analysis

Samples for which exsolution had been shown by pXRD measurements were mounted in phenolic resin, and the grain mounts were mechanically and chemo-mechanically polished to enable crystal orientation analysis by electron backscatter diffraction (EBSD). Crystal orientation analyses were performed using a FEI Quanta 3D FEG field-emission gun scanning electron microscope at the laboratory for field-emission scanning electron microscopy and focused ion beam applications at the FGGA, University of Vienna, Austria. This FIB-FESEM instrument is equipped with an Ametek EDAX Digiview 5 EBSD camera. Data collection was performed at electron beam settings of 15kV accelerating voltage and a probe current of ca. 4 nA at a working distance of 14 mm and 70° sample tilt. For each crystal, a single data point was collected with the Ametek EDAX OIM DC software v 7.3, applying a 2x2 camera binning, averaging over 8-10 image frames, and using image filters for static and dynamic background subtraction and contrast enhancement. Indexing is based on monoclinic (Laue symbol 2/m) reference structures for sample M (a = 8.357Å, b = 12.992 Å, c = 7.172 Å; $\alpha =\gamma =90^\circ$, $\beta =116.19^\circ$) and sample V (a=8.371Å, b=12.998 Å, c=7.169 Å; $\alpha =\gamma =90^\circ$, $\beta =116.13^\circ$). A minimum number of 3 and a maximum of 15-18 reflectors were considered for Hough based indexing at an interplanar angle tolerance of $2^\circ$. For each data point, Hough settings were adjusted and statistical indexing parameters were checked, in order to ensure that only statistically reliable orientation solutions were considered. Crystal orientations determined by EBSD were used as basis for orientation-specific focused ion beam (FIB) preparation of TEM foils and APT tips.

### Transmission electron microscopy

Electron transparent foils to be used for transmission electron microscopy were prepared by FIB nano-machining. Exsolution lamellae are sub-parallel to the Murchison plane, which has Miller indices in the range of ($\overline{8}$01) to ($\overline{6}$01) (Yund and Tullis 1983). Electron transparent foils oriented perpendicular to the [010] direction and thus perpendicular to the exsolution lamellae were extracted using the FIB technique on the same instrument as used for EBSD analysis. To this end, grains were selected, for which EBSD analysis revealed the [010] direction to have an angle of $1-5^\circ$ to the sample surface. FIB milling was done with a focused Ga ion beam at 30 kV accelerating voltage and stepwise lower ion probe currents from 65 to 3 nA. The foils were transferred to a Cu grid within the FIB-FESEM applying platinum deposition by FIB and using an Omniprobe 100.7 micromanipulator. Subsequently, the TEM foils were thinned to c. 100 nm thickness using stepwise lower ion probe currents of 1 nA to 30 pA. Final cleaning was performed at reduced ion beam accelerating voltage of 5kV/48pA and 2kV/27pA.

TEM analyses were done at the Faculty of Physics, University of Vienna (Austria) using a FEI Titan 80-300, which is equipped with a Schottky-field emission gun (FEG) and a spherical aberration (Cs) image-corrector. Bright-field (BF), dark-field (DF), high-resolution TEM (HRTEM) and selected area electron diffraction (SAED) imaging was performed under 80 kV acceleration voltage. The TEM point resolution with Cs correction is about 0.2 nm at 80 kV, which allows for atomic structure observation and analysis. An analytical double-tilt sample holder with a tilt range of $\pm 40^\circ$ around the first and $\pm 30^\circ$ around the second tilt axis was used for aligning the desired zone axis. Digital images were acquired with a Gatan 2k $\times$ 2k slow-scan charged coupled device camera.

### Atom probe tomography

For Madagascar sanidine the samples from experiments M-440-16 ($440^\circ \hbox {C}$ for 16 days), M-440-64, M-480-64, M-520-32, M-560-64; and for Volkesfeld sanidine the samples from experiments: V-440-32, V-440-128, V-480-128 were shown to contain exsolution lamellae by XRD and TEM analyses, and they were selected for chemical analysis by APT. For each of the selected samples, a series of 3 to 5 specimens were prepared for APT by in-situ lift out (Thompson et al. 2007). Grains with the exsolution lamellae sub-parallel to the sample surface were selected based on crystal orientation analysis by EBSD to ensure that the lamellae are oriented sub-perpendicular to the length axis of the APT tip. In this orientation the maximum number of lamellae are captured by the analyses, which provides the best composition statistics. Based on the notion that equilibrium can be assumed, when for a given annealing temperature lamellar compositions are equal for different annealing times, APT analysis was performed with different annealing times. Time series analyses were only done for the lowest temperature ($440^\circ \hbox {C}$) in order to determine the minimum required annealing time for the lamellae to attain equilibrium compositions. Since diffusion rates increase with temperature, once the annealing time needed for attainment of equilibrium compositions had been demonstrated for the lowest temperature samples, attainment of equilibrium can also be assumed for the higher temperature samples with similar annealing times.

After lift out, the specimens were sharpened using either a FEI Helios Nanolab 600i, a FEI Helios Nanolab 600 or a FEI Helios PFIB SEM, each equipped with a focused ion beam. All instruments are located at the Max-Planck-Institut für Eisenforschung GmbH (MPIE), Düsseldorf, Germany. Milling and sharpening was performed at 30 kV with a Xe-ion beam on the FEI Helios PFIB and a Ga-ion beam on the FEI Helios Nanolab 600i and 600. For the latter, sharpening was performed at cryogenic temperatures using the setup described in Rivas et al. (2020) to minimise implantation of Ga into the sample. For all instruments, a 5 kV beam was used in the last sharpening step to remove regions potentially damaged by the implantation of Ga ions.

APT analyses were performed on a Cameca Leap 5000 XR (MPIE) equipped with a reflectron-lens and a detector efficiency of $\sim$52%. The analyses were performed at 50 K in laser pulsing mode with a 355 nm laser wavelength, a laser pulse energy of 300-550 pJ, a detection rate of 0.5 to 1 ion detected for 100 pulses and a pulse frequency of 125 kHz.

The data were processed and 3D reconstructions were done with the commercial software package AP Suite 6.3. Reconstruction parameters were adjusted to reproduce the angles between exsolution lamellae and sample surface as previously determined by EBSD and to reproduce the lamellar thicknesses obtained for some samples from TEM.

The compositional profiles shown herein were computed from cylindrical regions of interest using the 1D compositional profile function with a fixed bin width of 0.1 nm and decomposed ion complexes. Since K and Na share the same site in the crystal structure and this study only focusses on their site fractions, the profiles were normalised so that Na+K=1 to minimise analytical artefacts. The presented profiles are K/(K+Na) and Na/(K+Na) in atomic proportions. Analytical parameters were adjusted for each specimen to ensure consistent field conditions and to minimise fractionation effects. Mass spectra (S6) show that Na and K only evaporated as $\hbox {K}^+$ and $\hbox {Na}^+$ and not as complex ions. To check for fractionation effects, the bulk composition obtained from APT was compared to the compositions obtained by EPMA. For all presented reconstructions, the deviations to EPMA results are $\le$2 mole %, depending mostly on whether an equal amount of K- and Na-lamellae were measured.

## Results

### Lamellar exsolution

The feature in pXRD data that is diagnostic for lamellar exsolution is the splitting of the ($\overline{2}$01) and the (111) reflections, due to the presence of two crystal lattices with significantly different dimensions in directions close to perpendicular to the lamellar interfaces. With decreasing temperatures, the split peaks become more widely separated, reflecting the increasing compositional differences between the Na-rich and the K-rich lamellae. The pXRD measurements performed on the samples that had been annealed for 64 days revealed split ($\overline{2}$01) and (111) reflections and thus the presence of exsolution lamellae at all temperatures given in Table 1 except for $560^\circ \hbox {C}$, where V did not show any sign of exsolution (Fig. S2). At all temperatures, the split peaks are more pronounced and sharper for M than for V (Fig. S2). The unit cell parameters of the exsolution lamellae are given in Table 2.

**Table 2:** Unit cell parameters of exsolution lamellae obtained by pXRD

Sample	Lamella	a [Å]	b [Å]	c [Å]	$\beta$ [°]
M-440-64	K	8.609 (3)	13.019(13)	7.226 (5)	116.30 (5)
	Na	8.076 (3)	12.886(11)	7.118 (6)	116.50 (7)
M-480-64	K	8.5010 (12)	13.017(2)	7.1994 (14)	116.139 (10)
	Na	8.2249 (12)	12.953(3)	7.100 (2)	116.132 (15)
M-520-64	K	8.4557 (14)	12.992(4)	7.197 (4)	116.32 (2)
	Na	8.249 (2)	12.974 (7)	7.115 (7)	116.13 (4)
M-560-64	K	8.3770 (16)	12.987 (3)	7.177 (3)	116.203 (17)
	Na	8.294 (2)	12.986 (7)	7.183 (5)	116.58 (3)
V-440-64	K	8.540 (3)	13.020 (3)	7.145 (3)	116.08 (3)
	Na	8.170 (2)	13.049 (5)	7.097 (2)	116.64 (2)
V-480-64	K	8.524 (7)	13.00 (3)	7.19 (2)	116.15 (10)
	Na	8.218 (5)	13.136 (19)	7.206 (17)	116.44 (9)
V-520-64	K	8.44 (4)	13.00 (4)	7.19 (3)	116.16 (13)
	Na	8.367 (10)	12.98 (3)	7.100 (12)	116.69 (16)

1$\sigma$ standard deviations are given in brackets; the 1$\sigma$ values apply to the last digits of the given values

**Fig. 1:**
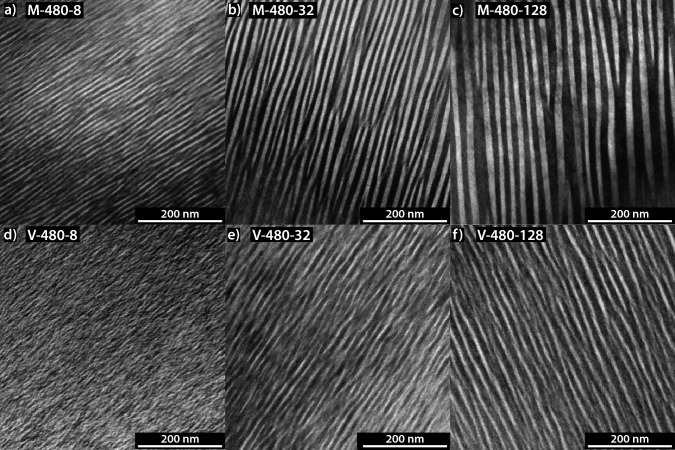
BF-TEM images viewed along the [010] direction for samples of M (**a**, **b** and **c**) and V (**d**, **e** and **f)** annealed for 8 (**a**, **d**), 32 (**b**, **e**) and 128 (**c**, **f**) days at $480^\circ \hbox {C}$. The orientation of the lamellae is close to $(\overline{8}01)$, hence the lamellae are viewed edge on in the figure. The lamellar coarsening is faster in M than in V. The contrast differences between the lamellae are more pronounced in M and show a sharper transition, while the transition in V is more gradual

**Fig. 2:**
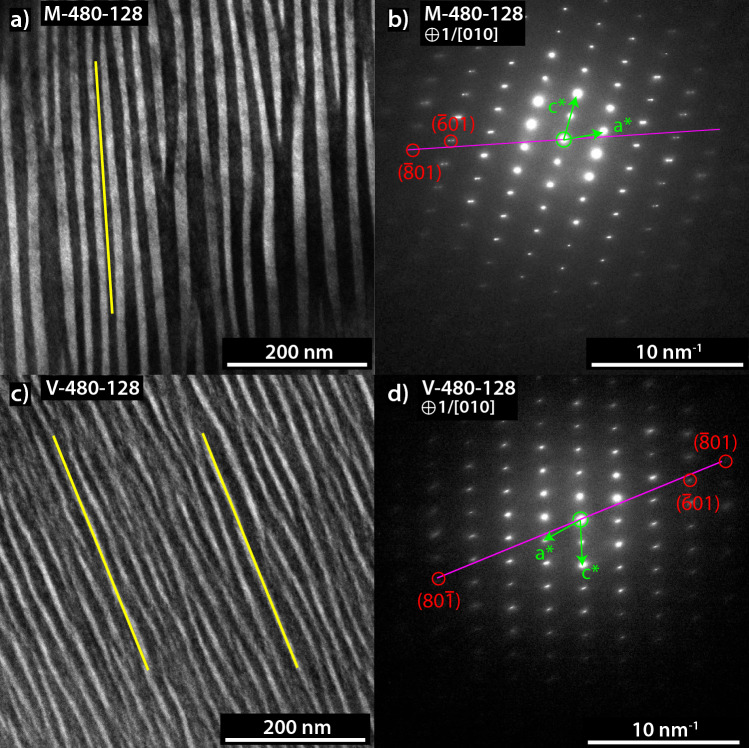
**a**, **c** BF-TEM images and **b**,**d** SAED images viewed along [010] for samples M-480-128 and V-480-128. The diffraction pattern of M (**b**) shows pronounced splitting of diffraction spots reflecting two distinct lattices. In a split diffraction spot the spot closer to the pattern centre corresponds to the K-rich lamellae, the more external spot corresponds to the Na-rich lamellae. The line connecting the spots of a split reflection (magenta lines) runs perpendicular to the lamellar interface (yellow lines). For directions that are parallel to the lamellar interfaces, no splitting of reflections occurs indicating that the crystal structures of the two lamellar types are coherent at the interface. For V, the SAED pattern (**d**) exhibits streaks perpendicular to the lamellar interfaces indicating coherency. The streaks probably arise due to a gradual transition across lamellar boundaries. The normal to the lamellar interface (magenta line) runs close to the $(\overline{8}01)$ diffraction spot indicating that the lamellae are oriented close to $(\overline{8}01)$

BF-TEM and SAED images of both M and V are shown in Fig. 1 and Fig. 2. Since all TEM images were acquired under [010] viewing direction, the lamellar interfaces are edge on, and the lamellar thicknesses are seen in true size. The BF-TEM images in Fig. 1 reveal that lamellar thickness increases with annealing time. For example, at 480°C for M lamellar thickness increases from $\sim$6 nm after annealing for 8 days to $\sim$9.5 nm after 32 days and to $\sim$15 nm after 128 days. For M, the lamellae are relatively straight with one lamellar type slightly thicker than the other one. In several places, the thicker lamellae show bifurcations, which correspond to the termination of one of the thinner lamellae. For given annealing temperature and run duration, the lamellae in V are generally thinner than those in M. For example, at $480^\circ \hbox {C}$ with only $\sim$4.7 nm after 8 days, $\sim$8 nm after 32 days and $\sim$10 nm after 128 days and the two lamellar types are not as sharply separated as in M but show more gradual transitions between the two lamellar types (Fig. 1).

SAED patterns acquired along the [010] zone axis show a distinct split of diffraction spots in all observed M samples (e.g. $(\overline{6}01)$ reflection in Fig. 2). Based on the fact that lattice constants increase with increasing *X*, out of a pair of split reflections the diffraction spot further from the pattern centre corresponds to the Na-rich lamellae, the diffraction spot closer to the pattern centre corresponds to the K-rich lamellae. The lamellar interfaces are perpendicular to the line connecting the split spots. Splitting of diffraction spots only occurs for diffraction spots corresponding to lattice planes that are sub-parallel to the lamellae or only enclose a small angle with the lamellae. The extent of diffraction spot splitting decreases with increasing angle that the corresponding lattice plane encloses with the lamellae, and diffraction spot splitting vanishes for diffraction spots corresponding to lattice planes that are oriented perpendicular to the exsolution lamellae. This indicates that the crystal structures of the Na-rich and the K-rich lamellae are coherent across the lamellar interfaces and both lamellae are elastically strained. The lattices Na-rich lamellae are dilated, and the lattices of the K-rich lamellae are shortened in the direction perpendicular to the lamellar interfaces. Coherency is also revealed by HR-TEM images, which show that the crystal structure is continuous across lamellar interfaces and is devoid of misfit dislocations (Fig. S4).

In the SAED patterns of V the reflections corresponding to lattice planes sub-parallel to the lamellar interfaces appear as streaks oriented perpendicular to the lamellar interfaces, but the diffraction spots are not clearly split (Fig. 2). The streaks cannot be explained by the fact, that the lamellae in V are thinner than in M, because M-480-32 shows clear splitting of the diffraction spots corresponding to lattice planes sub-parallel to the lamellar interface, whereas V-480-128 only shows streaks despite similar lamellar thicknesses. The streaks likely reflect a more gradual compositional transition between the Na-rich and the K-rich lamellae in V than in M. Only the analysis of Fourier transforms of small, several nanometers sized domains in HR images of V-480-128 revealed distinct diffraction spots for e.g. the $(\overline{6} 01)$ lattice planes, reflecting the different lattices of the Na-rich and the K-rich lamellae.

In all samples the normal to the interface plane is closely aligned with the ($\overline{8}$01) diffraction spot in the SAED patterns (Fig. 2), showing that the orientation of the lamellar interfaces is close to ($\overline{8}$01). Due to bending of the foils, the contrasts in BF and DF images vary, and it is mandatory for identification of the Na- or K-rich lamellae that the respective reflections are selected using the objective aperture. Aligning the objective aperture with the inner diffraction spot - the one closer to the pattern centre - of a split reflection, which corresponds to the K-rich lamellae, and with the outer diffraction spot, which corresponds to Na-rich lamellae, revealed that in sample M-520-32 the K-rich lamellae are thicker than the Na-rich lamellae. In addition, the differences in the thickness of the Na-rich and the K-rich lamellae and their compositions were directly analysed by APT, and, finally, volume ratios of the lamellae were calculated from the bulk composition measured by EPMA, the lamellar compositions obtained from APT and the respective molar volumes. All methods showed that the K-rich lamellae are slightly thicker than the Na-rich lamellae in M-440, M-480, M-520, V-440 and V-480 and have about double the thickness in M-560.

### Lamellar compositions

**Fig. 3:**
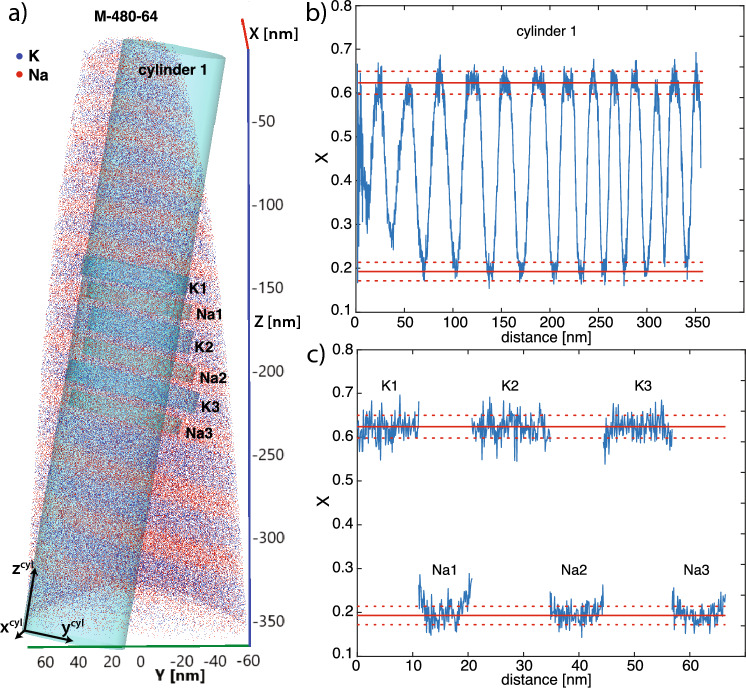
**a** Reconstructed APT dataset from sample M-480-64 with the cylindrical regions of interest chosen for extracting a compositional profile. **b** The compositions shown in the corresponding normalised profile were obtained from the atoms counted in the corresponding bin-width volume in the respective base-plane. c: Normalised profiles along the $z^{cyl}$ axes of the cylinders K1, Na1, K2, Na2, K3 and Na3 The averaged acquired compositions are shown as the red solid lines with 1$\sigma$ standard deviation indicated by the dotted red lines

Several cylindrical regions of interest were selected from each APT reconstruction to determine lamellar compositions and compositional profiles. The cylinders with base plane $\hbox {x}^{cyl}$-$\hbox {y}^{cyl}$ were oriented with their length axis ($\hbox {z}^{cyl}$) perpendicular to the lamellar interfaces. Compositional profiles were obtained in the same direction by integrating compositional data from each voxel over the respective $\hbox {x}^{cyl}$-$\hbox {y}^{cyl}$ plane for successive bins, where the bin width was 0.1 nm. An example of a APT reconstruction with corresponding compositional profiles is shown in Fig. 3.

The profiles reveal an alternation of K-rich and Na-rich lamellae. Most lamellae show compositional plateaus, which are quite uniform throughout a specimen. Locally, lamellar interfaces may not be exactly perpendicular to the length axis of the cylindrical region of interest. Then the respective $\hbox {x}^{cyl}$-$\hbox {y}^{cyl}$ plane cuts through both a K-rich and a Na-rich lamella producing a mixed signal. Such situations may arise at lamellar bifurcations. The compositions obtained from such locations were not considered for further analysis. For the determination of lamellar compositions, only composition plateaus that are furthest apart from each other and uniform over the entire specimen were considered (Fig. 3).

The lamellar compositions for each experiment are listed in Table 3 and are shown in Fig. 5 together with the 1$\sigma$ standard deviations as obtained from the scattering of the data in the plateau areas. As expected, for both feldspars the compositional difference between the Na-rich and the K-rich lamellae increases with decreasing temperature. For a given temperature, the miscibility gap appears to be smaller for V than for M. This is because at a given temperature, the Na-rich lamellae of V have more intermediate compositions than the Na-rich lamellae of M. For example, at $480^\circ \hbox {C}$ the Na-rich lamellae of M are characterised by $X = 0.193$ and and by $X = 0.294$ in V. The compositions of the K-rich lamellae are more similar for the two feldspars, but they are slightly more K-rich in M than in V. For example, at at $480^\circ \hbox {C}$ the K-rich lamellae are characterised by $X =0.624$ in M and by $X =0.587$ in V.

**Table 3:** Compositions of the Na-rich and the K-rich exsolution lamellae obtained from APT with 1$\sigma$ standard deviations

Sample	T [°C]	Time [days]	Na [X]	K [X]
M-440-16	440	16	0.157 ± 0.056	0.669 ± 0.055
M-440-64	440	64	0.139 ± 0.035	0.672 ± 0.040
M-480-64	480	64	0.193 ± 0.021	0.624 ± 0.026
M-520-32	520	32	0.228 ± 0.039	0.588 ± 0.040
M-560-64	560	64	0.324 ± 0.049	0.466 ± 0.050
V-440-32	440	32	0.318 ± 0.057	0.593 ± 0.048
V-440-128	440	128	0.250 ± 0.047	0.644 ± 0.055
V-480-128	480	128	0.294 ± 0.054	0.587 ± 0.059

### Theoretical calculation of the coherent solvus

Note that due to the strong compositional dependence of the lattice parameters of alkali feldspar solid solution, the Na-rich and the K-rich lamellae have different lattice parameters. Coherent lamellar intergrowth of Na-rich and K-rich alkali feldspar may be thought of as applying two specific stress-free chemical strains to the initial lattice of the compositionally homogeneous precursor feldspar. The lattices of both the Na-rich and the K-rich lamellae need then to be elastically strained so that the two lattices match at the lamellar interfaces This is where the elastic stresses, elastic strain and elastic strain energy come from.

The coherent solvus can be calculated by free energy minimisation based on a suitable thermodynamic mixing model that, in addition to the thermodynamic non-ideality of mixing, accounts for the elastic strain energy associated with coherent lamellar intergrowth of Na-rich and K-rich alkali feldspar. The basic concept is outlined in this section, for a detailed treatment the reader is referred to the work of Petrishcheva et al. (2023).

Due to reasons detailed below, the coherent solvus was only calculated for M for which $X_0=0.44$, where $X_0$ is the K-mole fractions of the homogeneous precursor phase. The compositional dependence of the lattice parameters was taken from Heuser et al. (2024) and was fitted by polynomial functions, see Equation (S.3). The thermodynamic non-ideality of Na and K mixing was encoded in a sub-regular two-parameter Margules type mixing model with Margules parameters $W_{\textrm{gK}}$ and $W_{\textrm{gNa}}$ and with the temperature dependence expressed as $W_g=W_h-W_sT$ given by Heuser et al. (2024):

$$\begin{aligned} W_{\textrm{gK}}= & {} 19754\,\hbox {Jmol}^{-1} - 2.33T\,\hbox {Jmol}^{-1}\hbox {K}^{-1} \\ W_{\textrm{gNa}}= & {} 14916\,\hbox {Jmol}^{-1} - 3.55T\,\hbox {Jmol}^{-1}\hbox {K}^{-1} \end{aligned}$$

**Fig. 4:**
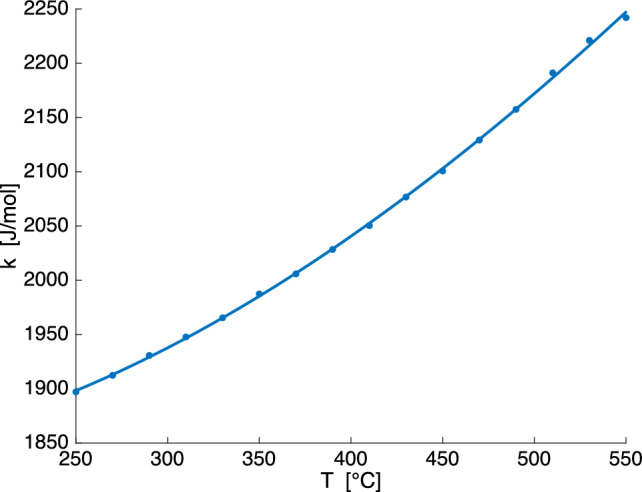
$k(T,X_0)$ obtained from an iterative procedure following Petrishcheva et al. (2023) for $X_0=0.44$ as used for calculating the elastic strain energy from ${\mathcal {E}}=k (X - X_0)^2$

The elastic strain energy may be written as (Cahn 1962)

1 $$\begin{aligned} {\mathcal {E}}=k (X - X_0)^2, \end{aligned}$$

where *X* and $X_0$ are the K-mole fractions of one of the segregated lamellae and of the homogeneous precursor phase, respectively. The parameter $k=k(T,X_0)$ depends on temperature *T* and on the composition $X_0$ of the homogeneous precursor feldspar. For calculation of $k=k(T,X_0)$ we use an iterative procedure (Petrishcheva et al. 2023):

(0) We choose a temperature.
(1) We use the purely chemical expression for the Gibbs energy to calculate *g*(*X*, *T*), and obtain the binodal compositions by Gibbs energy minimisation (common tangent construction). We follow the decomposition process up to the binodal points, and calculate the accumulated elastic strain energy.
(2) The calculated elastic strain energy yields a new expression for the total (chemical+elastic) Gibbs energy. The binodal compositions are then recalculated.
(3) The decomposition process is considered once again, now up to the new-calculated binodal points, and a more accurate expression for the elastic strain energy is obtained.
(4) Steps (2) and (3) are repeated until the binodal points and elastic strain energy converge to their final values, which yields $k(T,X_0)$.
(5) We then return to step (0) and chose another *T*. Altogether we consider temperatures within the interval 250 °C ≤ *T* ≤ 560 °C with steps of 20 °C. The resulting $k(T, X_0)$ is shown in Fig. 4
(6) After the contribution of the elastic strain energy to the Gibbs energy of stably coexisting coherently intergrwon layers of Na-rich and K-rich alkali feldspar is quantified, we calculate the coherent solvus.

The outlined calculation yields the coefficient $k(T,X_0)$ needed for calculating the elastic strain energy contribution to the free energy according to Eq. (1), see Fig. 4. The elastic constants were taken from Haussühl (1993) as specified in Table 4.

**Fig. 5:**
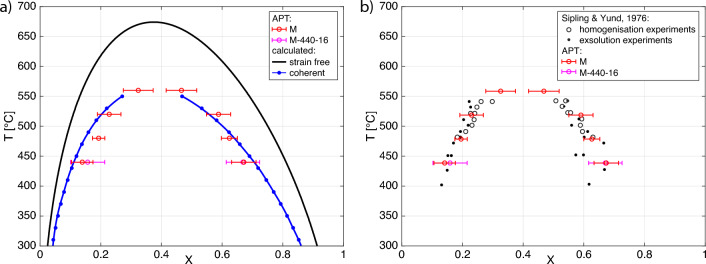
**a** Composition-temperature diagram showing the compositions obtained from APT analyses of coherently intergrown Na-rich and K-rich lamellae produced from exsolution experiments with 1$\sigma$ standard deviation (red). The calculated strain free and coherent solvus are shown as solid black and blue lines, respectively. **b** Lamellar compositions from Sipling and Yund (1976) (black open circles and black dots), for reference the ATP data shown in **a** are reproduced

Combining the Gibbs energy of the strain free alkali feldspar solid solution and the elastic strain energy contribution provides the basis for iterative calculation of the binodal compositions for coherent lamellar intergrowth at a given temperature. Performing this calculation at different temperatures yields the coherent solvus, see the blue line in Fig. 5a. The calculated coherent solvus agrees very well with the experimentally determined phase relations. The procedure of calculating the coherent solvus can be applied to any feldspar provided that (i) the initial composition, (ii) the Gibbs energy of the strain free solid solution, and (iii) the stiffness coefficients are available.

**Table 4:** Stiffness coefficients in GPa from Waeselmann et al. (2016) (based on data from Haussühl (1993)) rotated to $x_1 \parallel {\textbf{a}}$, $x_2 \parallel {\textbf{b}}$, $x_3 \parallel {\textbf{c}}^{\star }$

	$c_{i,1}$	$c_{i,2}$	$c_{i,3}$	$c_{i,4}$	$c_{i,5}$	$c_{i,6}$
$c_{1,j}$	68.7	49.2	38.5	0	$-$2.5	0
$c_{2,j}$	49.2	176.8	15.4	0	1.2	0
$c_{3,j}$	38.5	15.4	134.7	0	$-$29.7	0
$c_{4,j}$	0	0	0	13.5	0	$-$1.1
$c_{5,j}$	$-$2.5	1.2	$-$29.7	0	30.5	0
$c_{6,j}$	0	0	0	$-$1.1	0	39.3

## Discussion

### Lamellar compositions

To determine whether the compositions obtained from APT measurements of the Na- and K-rich lamellae correspond to binodal compositions, it must be ensured that thermodynamic equilibrium between the Na- and K-rich lamellae was attained. Thus, for each feldspar two samples, both annealed at the lowest temperature for different run durations, were analysed by APT. Samples M-440-16 (16 days) and M-440-64 (64 days) yield compositions for the Na-rich and the K-rich lamellae that are identical within error for the two run durations (Tab. 3) indicating that equilibrium compositions were attained at $440^\circ \hbox {C}$ after 16 days. It may safely be assumed that equilibrium compositions were also attained in experiments at $440^\circ \hbox {C}$ with longer run durations and in experiments at higher temperatures and with similar or longer run durations. The corresponding lamellar compositions thus define points on the coherent solvus.

Lamellar compositions obtained for V-440-32 (32 days) and V-440-128 (128 days) differ, however, significantly (Tab. 3) indicating that in V equilibrium compositions were not yet attained after annealing at $440^\circ \hbox {C}$ for 32 days and possibly were not even reached after 128 days. Moreover, for all temperatures the lamellae in V generally show gradual compositional transitions between the Na-rich and the K-rich lamellae. The lamellae lack compositional plateaus, so that their characteristic compositions cannot be determined with sufficient accuracy.

Therefore, only the lamellar compositions of M were considered as binodal compositions defining points on the coherent solvus, and further treatment is focused on M. The measured coherent solvus for M lies well below the solvus curve calculated for strain free phase equilibria, where at intermediate compositions the temperature difference between the measured coherent and the calculated strain free solvus curve is about $100^\circ \hbox {C}$ (Fig. 5a), The measured coherent solvus for M coincides reasonably well with the lamellar compositions determined by Sipling and Yund (1976), which were derived from the lattice parameters determined for experimentally exsolved high sanidine, using the compositional dependence of the lattice parameters given by Orville (1967) and the strain correction after Tullis (1975) (Fig. 5b).

### Comparison of observed and calculated coherent solvus

Figure 5a shows that the calculated coherent solvus reproduces the experimentally determined lamellar compositions very well within the error of the APT measurements. It is known and was confirmed by our pXRD measurements that disordered Na-rich alkali feldspar is metrically triclinic with space group $C\overline{1}$, while more K-rich disordered alkali feldspar is monoclinic with space group *C*2/*m*. According to our pXRD measurements this transition occurs at $0.33<X<0.44$ for M and at $0.21<X<0.37$ for V (Heuser et al. 2024). In this compositional range transformation weakening may be expected, but the extent of such weakening is not known. Due to the lack of data on the compositional dependence of the elastic constants at intermediate compositions, a constant stiffness tensor was used for calculating the elastic strain energy in coherent lamellar intergrowth (Petrishcheva et al. 2023). To test for the potential influence of different elastic constants, solvi were also calculated with the stiffness tensors given by Brown et al. (2006) and Waeselmann et al. (2016) for K- and Na-rich alkali feldspars. The resulting solvi are shown in Fig. S7 in the Supplementary Information, which reveals that the influence of different elastic constants is minute, and there are only minor differences in the sizes and positions of the coherent solvi obtained by using different sets of stiffness coefficients. The largest influence is seen for the solvus calculated based on the stiffness coefficients of albite, which are higher then the stiffness coefficients for K-rich alkali feldspar and produce the lowest solvus, accordingly.

For the solvi calculated based on all other sets of stiffness coefficients the match between experimentally determined lamellar compositions and the calculated coherent solvus is very satisfactory. This indicates that the thermodynamic non-ideality of disordered alkali feldspar solid solution taken from Heuser et al. (2024) combined with the elastic strain energy contribution to the free energy as calculated after Petrishcheva et al. (2023) provide a sound basis for the thermodynamic analysis of coherent exsolution in alkali feldspar. Understanding phase equilibrium in coherent lamellar intergrowth of Na-rich and K-rich alkali feldspar is fundamental for interpreting the compositions of the different phases in perthites. In particular, it is a pre-requisite for modelling the compositional evolution of coherent perthites during cooling using diffuse interface models (Petrishcheva and Abart 2009, 2012), which so far has only been done for perthites that lost coherency (Abart et al. 2009a, b), and only now has become feasible for perthites that preserved coherency across lamellar boundaries.

### Differences in exsolution behaviour between M and V feldspars

Based on the differences in lamellar thicknesses and in the characteristics of the compositional transitions between the Na-rich and K-rich lamellae, M and V are inferred to behave differently during exsolution. The small differences in the state of Al-Si order between the two feldspars cannot explain their different exsolution behaviour. Neither can the thermodynamics of Na-K mixing be responsible, as both feldspars have been shown to produce similar Na-K partitioning with NaCl-KCl melt (Heuser et al. 2024). The two feldspars differ, however, in that V contains significantly more Ba than M (see Table S1). In V, 1 out of 77 alkali sites is occupied by $\hbox {Ba}^{2+}$, which is immobile during Na-K exchange between NaCl-KCl salt melt and sanidine (Neusser et al. 2012). Charge balance requires that each $\hbox {Ba}^{2+}$ ion is accompanied by an extra $\hbox {Al}^{3+}$ in the tetrahedral framework. Ba-Na or Ba-K interdiffusion thus necessitates concomitant Si-Al interdiffusion, which is, however, exceedingly sluggish (Grove et al. 1984; Cherniak 2003). As a consequence, Ba diffusion is slow (Cherniak 2002), which may also slow down Na-K interdiffusion and thus chemical segregation of the Na-rich and K-rich lamellae as well as lamellar coarsening in V as compared to M. Moreover, the presence of Ba in V may also impede exsolution due to a thermodynamic effect. The ionic radius of $\hbox {Ba}^{2+}$ (0.135 nm) is much closer to the ionic radius of $\hbox {K}^{+}$ (0.135 nm) than to the ionic radius of $\hbox {Na}^{+}$ (0.95 nm), and in equilibrium between two alkali feldspars with different K mole fractions, Ba is preferentially partitioned into the K-rich phase (Cherniak 2002). Due to exceedingly sluggish diffusion, $\hbox {Ba}^{2+}$ cannot follow $\hbox {K}^{+}$ during segregation of Na and K producing non-equilibrium partitioning of Ba between the Na-rich and the K-rich lamellae (Fig. S5). This makes exsolution in the Ba-bearing system V energetically less favourable than in the Ba-free system M. Both the kinetic and the thermodynamic effect tend to impede or suppress exsolution and serve as a qualitative explanation for the different exsolution behaviour of V and M.

## Summary and conclusions

Two homogeneous gem-quality high sanidines from Madagascar and from Volkesfeld (Eifel, Germany) with intermediate Na-K compositions were experimentally exsolved between 440°C and 560°C at close to ambient pressure. The compositions of the 10 to several 10 s of nm thick relatively more Na-rich and relatively more K-rich exsolution lamellae were measured with APT. Splitting of the reflections corresponding to lattice planes oriented sub-parallel to the lamellar interfaces in pXRD data and of the corresponding diffraction spots in TEM SAED indicates coherent intergrowth. Time series experiments revealed that only the lamellar compositions obtained for the Madagascar feldspar correspond to binodal compositions defining points on the coherent solvus, whereas the exsolution lamellae in Volkesfeld sanidine do not appear to have reached equilibrium compositions. The difference in exsolution behaviour between the Madagascar and Volkesfeld sanidines is ascribed to the comparatively high Ba content of the latter. The observed Ba mole fraction of $\approx 0.01$ on the alkali site tends to impede Na-K interdiffusion and the notorious immobility of Ba leads to non-equilibrium partitioning of Ba between the exsolved Na-rich and K-rich lamellae, which makes exsolution energetically less favourable than exsolution in Ba free alkali feldspar.

The coherent solvus determined from the compositions of the experimentally produced exsolution lamellae in Madagascar sanidine is well reproduced by the coherent solvus calculated from a thermodynamic mixing model that had been calibrated on the same feldspar and also accounts for the elastic strain energy associated with lamellar intergrowth. This shows that the thermodynamic mixing properties of Madagascar sanidine as well as the elastic strain energy are well described by our model. Knowledge of the coherent solvus of alkali feldspar is fundamental for interpreting the lamellar compositions in perthites that preserved coherent intergrowth of the exsolution lamellae.

## Supplementary Information

Below is the link to the electronic supplementary material.

Supplementary file 1 (pdf 9077 KB)

## Data Availability

All relevant data can be found in the supplementary information.

## Footnotes

1 Trade name “Madagascar Orthoclase”
