# Supplementary material for: Coherent solvus of disordered alkali feldspar: experiment, atom probe tomography and thermodynamic model
Source: Contrib Mineral Petrol. 2024 Jun 6;179(6):68. doi: 10.1007/s00410-024-02150-z (PMC11636987; doi:10.1007/s00410-024-02150-z)
Supplement: Supplementary file 1 — (pdf 9077 KB) [file 410_2024_2150_MOESM1_ESM.pdf]

Supplementary Information

pXRD measurements

For calculating chemical strains the unit cell parameters given by Heuser et al. (2024) were used. For M cubic polynomials were fitted to the measured compositional dependence of the b, c and β parameters, and a quadratic polynomial was fitted to the measured compositional dependence of the a parameter (see Fig. S1):

$$\begin{aligned} a_M =& -0.0243X^2 + 0.5038X + 8.1429 \\ b_M =& 0.3112X^3 - 0.7163X^2 + 0.5882X + 12.8356 \\ c_M =& 0.0751X^3 - 0.1723X^2 + 0.1716X + 7.1260 \\ \beta_M =& 1.0573X^3 - 0.9959X^2 - 0.5206X + 116.5243\ . \end{aligned} \tag{S.2}$$

For V, unit cell parameters are only available for a smaller compositional range than for M in Heuser et al. (2024), and polynomial fits do not give reasonable results for compositions outside of that compositional range for b, c and β. Hence, the shapes of the Analbite - High Sanidine curves given by Kroll and Ribbe (1987) were used for b, c and β and only the intercept was fitted to the measured lattice parameters of V. a was fitted with a quadratic polynomial. The resulting relations for V are

$$\begin{aligned} a_V =& -0.0787X^2 + 0.5646X + 8.1366 \\ b_V =& 0.1187X^3 - 0.3892X^2 + 0.4356X + 12.8649 \\ c_V =& 0.0988X^3 - 0.2282X^2 + 0.2033X + 7.1145 \\ \beta_V =& 0.9248X^3 - 0.8906X^2 - 0.4602X + 116.4409\ . \end{aligned} \tag{S.3}$$

Excerpts from diffraction profiles for $13° < 2\theta < 33°$ for M and for $19° < 2\theta < 34°$ for V are depicted in Fig. S2, showing the split peaks for the $(20\bar{1})$ and (111) reflection bands wich indicate exsolution.

Electron Probe Microanalysis and high-resolution Transmission Electron Microscopy

Major element compositions for the starting materials determined with EPMA are given in Tab. S1. High contrast BSE images (Fig. S3) and line measurements through grains showed that homogeneous compositions were reached throughout the grains after shifting the compositions through cation-exchange with NaCl-KCl melt at 900°C for 35 days. The

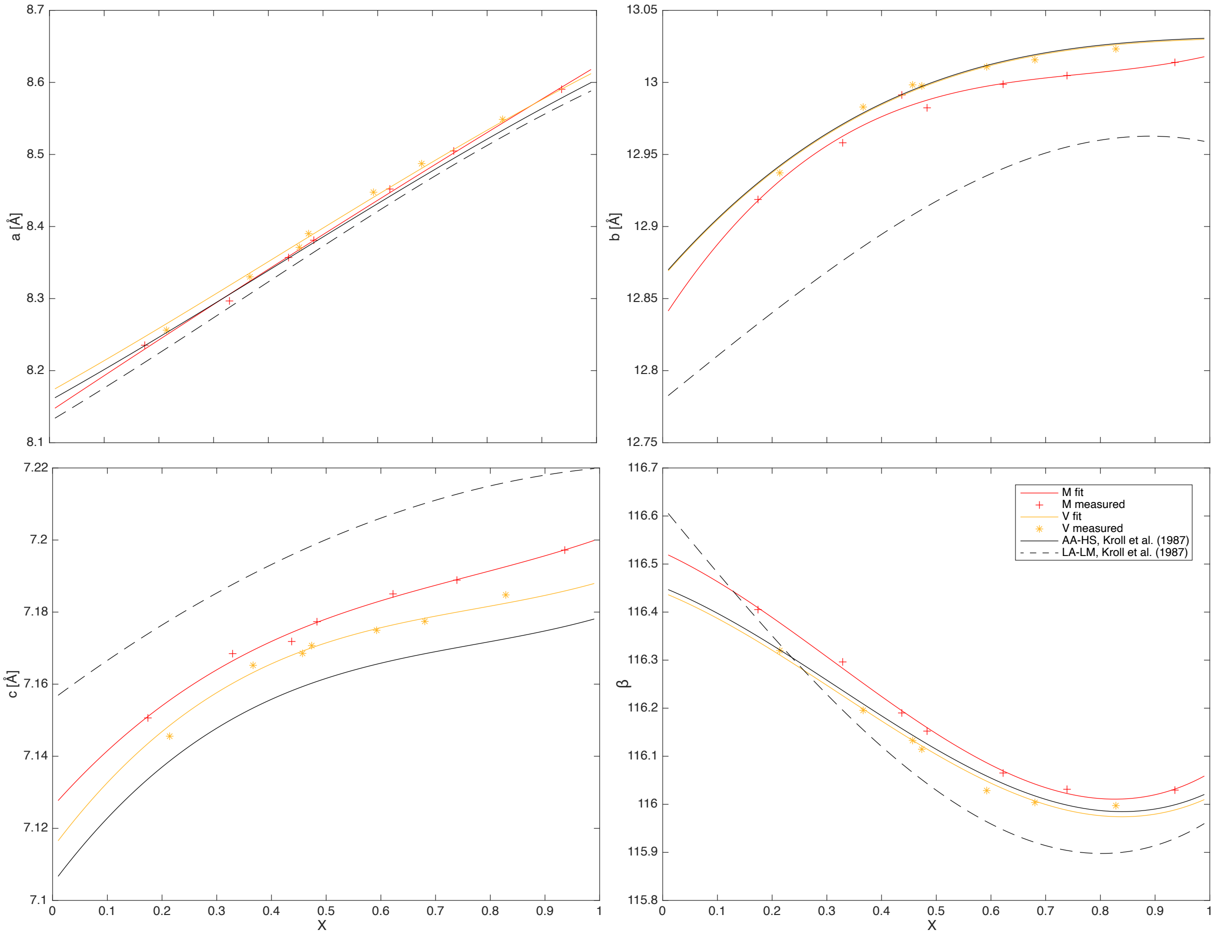

Figure S1: Variation of unit cell parameters a, b, c and β with X for M (red) and V (yellow); data points were obtained from the pXRD measurements of Heuser et al. (2024); equations eqs. (S.2) and (S.3) were used for fitting. For comparison, the curves for the analbite - high sanidine series (solid line) and the low albite - low microcline series (dashed line) from Kroll and Ribbe (1987) are shown in black.

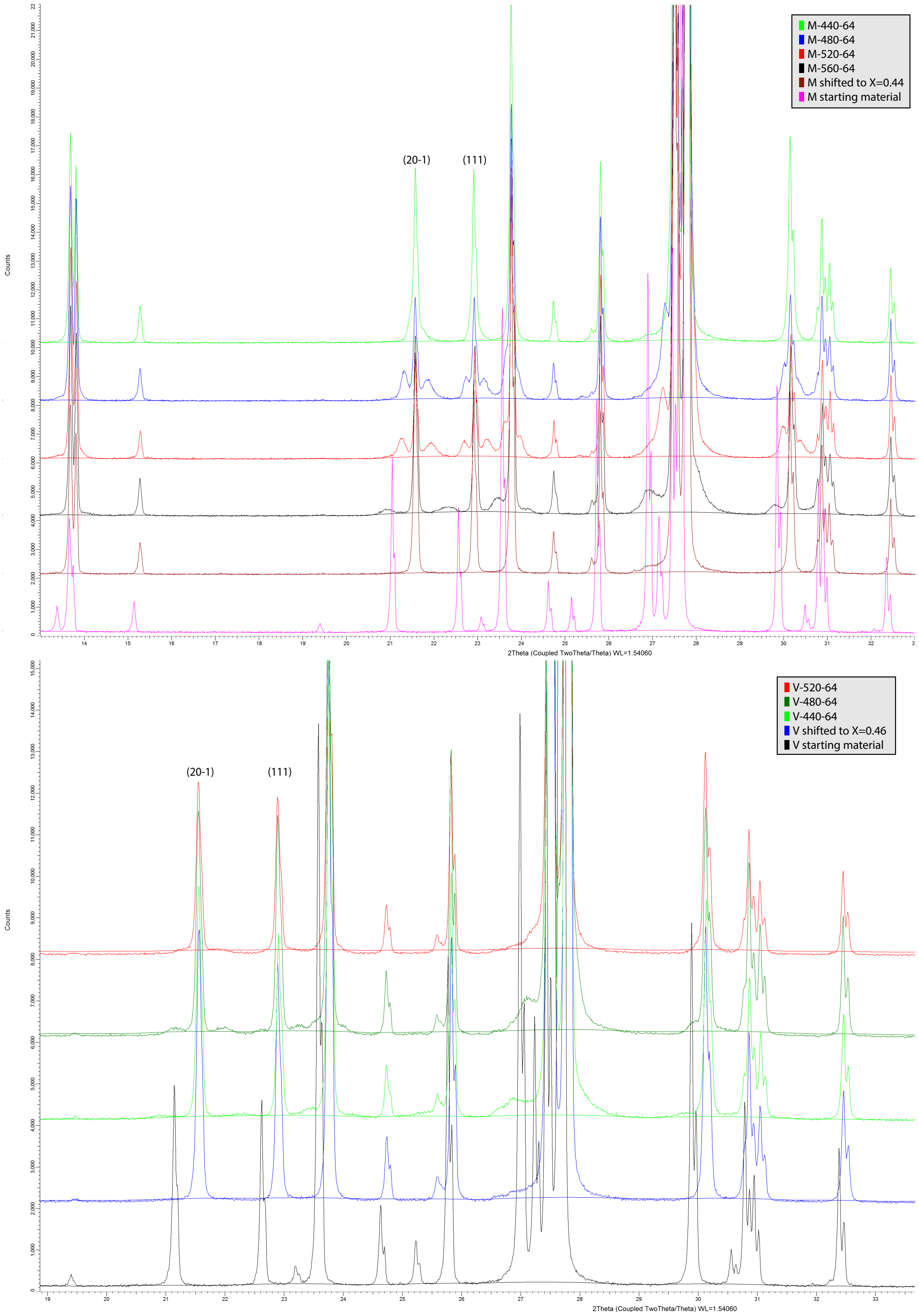

Figure S2: Diffraction profiles obtained from pXRD measurements of M and V samples as listed in the legends. The material that was changed to more Na-rich compositions is clearly shifted towards higher 2Θ values. The exsolved material shows split peaks corresponding to the exsolution lamellae for the (20-1) and the (111) reflection band.

target compositions and required amounts of feldspar and salt for the Na-K-exchange experiments are given in Tab. S2.

In addition to BF and SAED TEM images, HR-TEM images were taken showing a continuous lattice throughout the lamellar intergrowth (Fig. S4).

Table S1: Compositions of the alkali feldspar starting materials in wt% oxide for Na_2O, K_2O, Al_2O_3, SiO_2, CaO and Fe_2O_3 as obtained from EPMA and in ppm for Ba, Rb and Sr, which were measured with ICP-MS. The sum formulae were calculated based on 5 cations. Charge balance and spectroscopic analysis (Coombs, 1954; Hofmeister and Rossman, 1984) show that Fe is present as Fe^{3+} in M.

wt% ox	M	V	apfu	M	V
Na_2O	0.578	1.737	Na	0.052	0.156
K_2O	15.802	14.052	K	0.936	0.828
Al_2O_3	17.242	18.960	Al	0.944	1.033
SiO_2	65.028	64.002	Si	3.020	2.957
CaO	0.000	0.000	Ca	0.000	0.000
Fe_2O_3	1.271	0.213	Fe	0.044	0.007
Ba [ppm]	765	6559	Ba	0.002	0.013
Rb [ppm]	162	172	Rb	0.001	0.001
Sr [ppm]	49	1355	Sr	0.000	0.004
Total	100.019	99.773	Total	4.999	4.999

ICP-MS measurement

The feldspar powders were first dried at 105 °C for 2 h. Then, about 40 mg powder was dissolved in a Teflon beaker using a mixture of concentrated nitric (HNO_3) and hydrofluoric (HF) acid with a volumetric ratio of 1:2. The uncapped beakers were heated to 100 °C for 30 minutes on a hot plate, then tightly capped and heated to 180 °C for at least 48 hours. Afterwards, the acid was evaporated and the residue was dissolved in 15N HNO_3, then evaporated and dissolved in 12N HCl, evaporated again and dissolved in 2 mL of 7.5 N HNO_3 and finally it was diluted ∼1500 times. Analytical results for the standards GSP-2, JR-2, BCR-2 and BHVO-2 were within the specified uncertainties. The detection limits are at 0.05 μg/g. The concentrations of all elements measured by ICP-MS are given in Tab. S3.

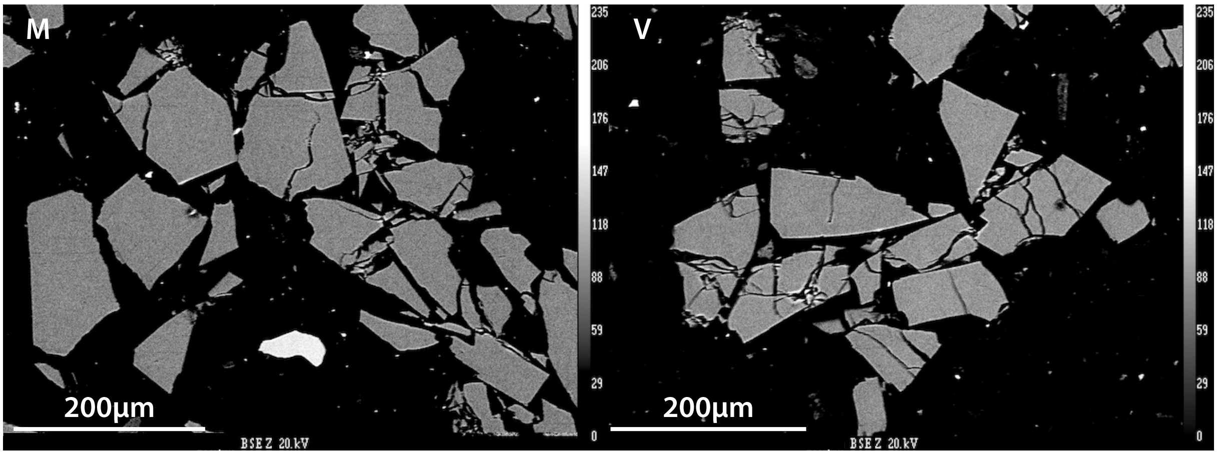

Figure S3: High contrast BSE images of mounted and polished powder of M (left) and V (right) after it has been shifted to intermediate compositions via cation exchange with NaCl-KCl-melt. The grey shade is very homogeneous within the samples indicating uniform composition throughout the grains.

Table S2: Target compositions and target amounts of feldspar powder and salt for the Na-K-exchange experiment.

	M	V
X_{fsp}	0.440	0.440
X_{salt}	0.226	0.231
n_{fsp} [mol]	0.007	0.007
n_{KCl} [mol]	0.061	0.061
n_{NaCl} [mol]	0.207	0.203
m_{fsp} [g]	1.868	1.830
m_{KCl} [g]	4.517	4.550
m_{NaCl} [g]	12.122	11.853

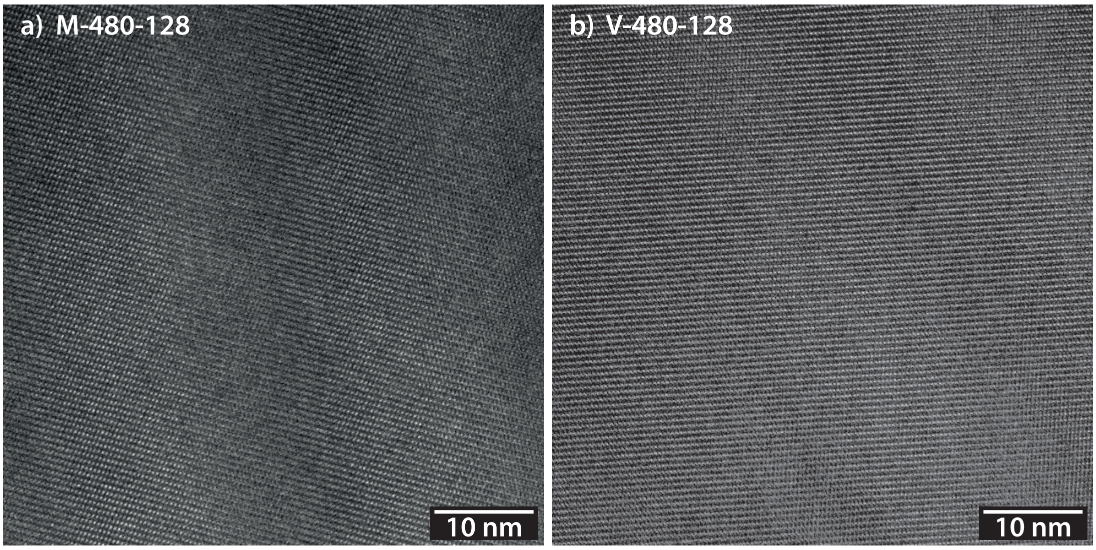

Figure S4: HR-TEM images viewed along the [010] direction for samples M-480-128 (a) and V-480-128 (b).

Table S3: Minor and trace element concentrations [mg/kg] in the starting materials M (Madagascar sanidine) and V (Volkesfeld sanidine) measured by ICP-MS

[mg/kg]	M	V	[mg/kg]	M	V	[mg/kg]	M	V
Li	0.27	0.87	Rb	162	172	Eu	0.08	0.64
Be	8.55	0.24	Sr	48.90	1355	Gd	0.01	0.05
B	32.00	1.49	Y	0.09	0.25	Tb	0.00	0.00
Sc	0.10	0.02	Zr	4.41	5.24	Dy	0.00	0.01
Ti	233	97.00	Nb	0.09	0.33	Ho	0.00	0.00
V	0.36	0.16	Mo	0.53	0.46	Er	0.00	0.00
Cr	9.89	9.25	Ag	0.02	0.04	Tm	0.00	0.00
Mn	7.61	6.80	Cd	0.08	0.01	Yb	0.00	0.00
Fe	8342	1522	Sn	0.26	0.33	Lu	0.00	0.00
Co	0.19	0.33	Sb	0.06	0.05	Hf	0.02	0.03
Ni	11.60	11.60	Cs	0.17	0.39	Ta	0.03	0.15
Cu	7.95	12.10	Ba	765	6559	W	11.00	5.91
Zn	12.90	8.56	La	0.08	0.47	Tl	0.61	0.20
Ga	20.80	17.10	Ce	0.31	0.89	Pb	1.34	2.91
Ge	0.01	0.01	Pr	0.01	0.04	Bi	0.04	0.01
As	0.10	0.12	Nd	0.09	0.15	Th	0.04	0.06
Se	0.00	0.01	Sm	0.01	0.04	U	0.01	0.02

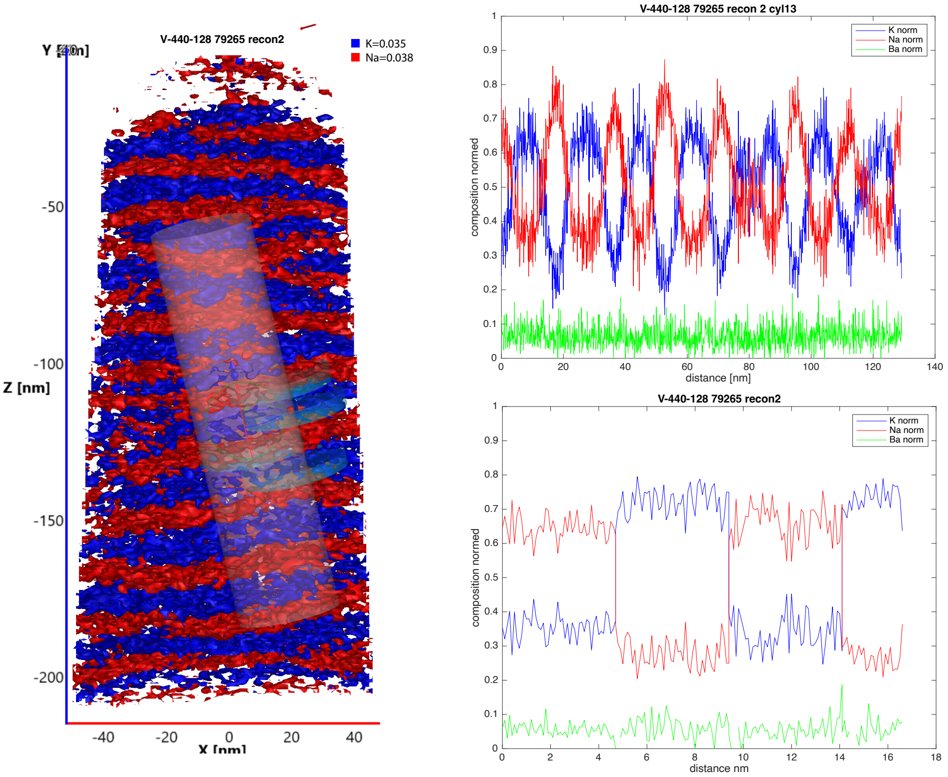

Figure S5: A reconstructed APT dataset from sample V-440-128 shown with the cylinders that were selected to extract composition profiles and the corresponding profiles for K, Na and Ba. Ba-peaks were only detected in V-440-32 and V-440-128.

APT

APT analyses provide full 3D reconstructions of the main element spatial distribution in the measured samples. Since this work focuses on the exsolution of Na and K, only Na- and K-maps are shown and discussed in the main text. However, exsolution could affect the distribution of minor elements such as Ba, which is more easily accommodated in K-rich feldspars (Cherniak, 2002). Ba-peaks were only detected in V-440-32 and V-440-128. In both samples, Ba is uniformly distributed and did not segregate during exsolution, which confirms that Ba ions move much more slowly in the feldspar lattice than K or Na ions. A reconstructed APT dataset for V-440-128 is shown in Fig. S5 together with composition profiles for Na, K and Ba, that were extracted from the cylinders shown in the reconstruction. A mass spectrum from an APT- specimen prepared from M-480-64 is shown in Fig. S6.

Comparison of solvi calculated from different stiffness tensors

To calculate the elastic strain energy associated with coherent exsolution, the elastic properties of disordered alkali feldspars with X=0.89 were taken from Haussühl (1993).

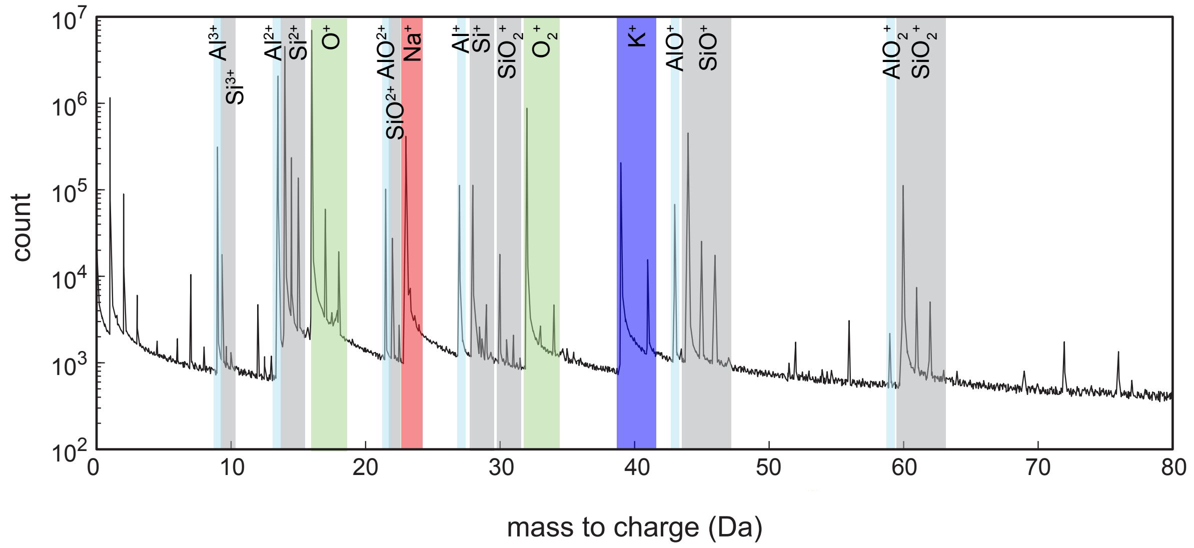

Figure S6: APT mass spectrum taken from a specimen prepared from M-480-64, where the peaks are indicated with the assigned ions.

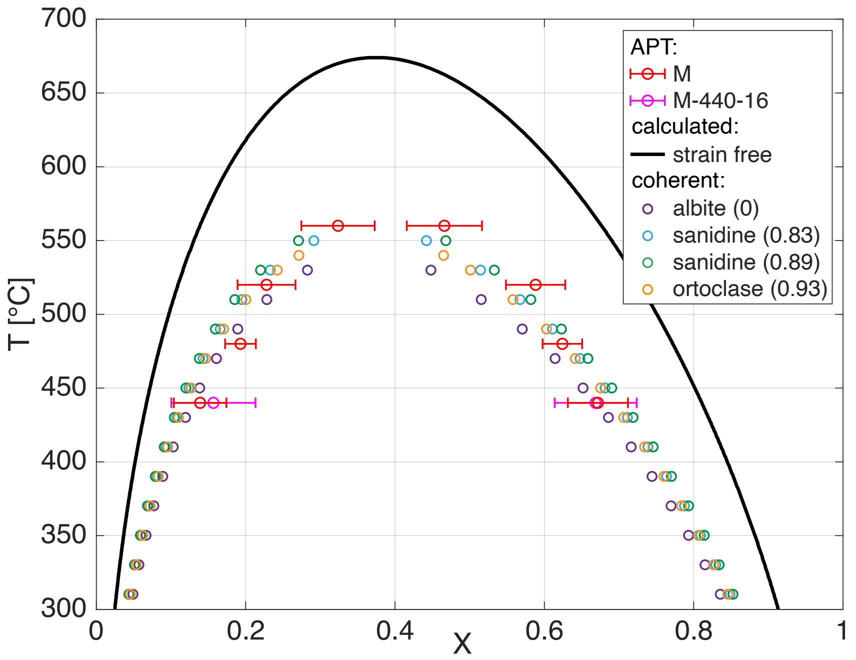

Figure S7: Strain free and coherent solvi plotted together with the measured APT-data. The coherent solvi were calculated with different elastic parameters that were obtained for different alkali feldspars. Violett: low albite (X=0) from Brown et al. (2006); light blue: sanidine (X=0.83) from Waeselmann et al. (2016); green: sanidine (X=0.89) given by Haussühl (1993); orange: orthoclase (X=0.93) from Waeselmann et al. (2016).

Table S4: Stiffness coefficients in GPa taken from Haussühl (1993), Brown et al. (2006) and Waeselmann et al. (2016) rotated to $x_1 \parallel \mathbf{a}$, $x_2 \parallel \mathbf{b}$, $x_3 \parallel \mathbf{c}^\star$,

	low albite (X=0) Brown et al. (2006)	sanidine (X=0.83) Waeselmannn et al. (2016)	sanidine (X=0.89) Haussuehl (1993)	orthoclase (X=0.93) Waeselmannn et al. (2016)
c_{11}	79.4	68.1	68.7	68.1
c_{12}	34.6	43.8	49.2	46.8
c_{13}	38.7	36.9	38.5	34.6
c_{15}	-9.2	-2.3	-2.5	-1.5
c_{22}	183.5	176.2	176.8	181.2
c_{23}	4.9	12.1	15.4	14.2
c_{25}	6.5	5.0	1.1	-0.1
c_{33}	154.2	136.1	134.7	138.8
c_{35}	-31.1	-29.2	-29.7	-28.3
c_{44}	20.9	12.9	13.5	14.3
c_{46}	-1.0	-1.5	-1.1	-2.4
c_{55}	34.7	32.3	30.5	29.0
c_{66}	37.5	39.7	39.3	39.9

To check for the influence of the elastic properties, the coherent solvus was also calculated using elastic constants for low albite given by Brown et al. (2006) and for orthoclase and sanidine given by Waeselmann et al. (2016) (Fig. S7). To this end, the stiffness tensors were rotated to $x_1 \parallel \mathbf{a}$, $x_2 \parallel \mathbf{b}$, $x_3 \parallel \mathbf{c}^\star$. The resulting stiffness coefficients are given in GPa in Tab. S4. It is seen from Fig. S7 that the influence of different elastic constants is modest, and there are only minor differences in the sizes and positions of the coherent solvi obtained by using different sets of stiffness coefficients.
